# Diagnostic Yield and Outcomes of Small Bowel Capsule Endoscopy in Patients with Small Bowel Bleeding Receiving Antithrombotics

**DOI:** 10.3390/diagnostics14131361

**Published:** 2024-06-27

**Authors:** Nikos Viazis, Dimitris Christodoulou, Vasilis Papastergiou, Konstantinos Mousourakis, Dimitra Kozompoli, Giannis Stasinos, Konstantina Dimopoulou, Periklis Apostolopoulos, Fotios Fousekis, Christos Liatsos, Nikolaos Kyriakos, Theodoros Argyropoulos, George Tribonias

**Affiliations:** 1 Gastroenterology Department, “Evangelismos” General Hospital, 16121 Athens, Greece; vasi.pap@hotmail.com (V.P.); konstantinosmousourakis@gmail.com (K.M.); dimitraskozompoli@gmail.com (D.K.); 2 Department of Gastroenterology and Hepatology, University Hospital of Ioannina, University of Ioannina, 45110 Ioannina, Greece; dchristodoulou@gmail.com (D.C.); fotisfous@gmail.com (F.F.); 3 Gastroenterology Department, NIMTS, 19010 Kalivia, Greece; g.tribonias@gmail.com (G.S.); periklisapo@yahoo.com (P.A.); 4 Gastroenterology Department, Red Cross Hospital, 11526 Athens, Greece; conu_med@hotmail.com (K.D.); argyropoulostheodoros@gmail.com (T.A.); g.tribonias@gmai.com (G.T.); 5 Gastroenterology Department, 401 Military Hospital of Athens, 11525 Athens, Greece; cliatsos@yahoo.com (C.L.); nikos_kiriakos@yahoo.gr (N.K.)

**Keywords:** small bowel, capsule endoscopy, bleeding, antithrombotic, antiplatelet therapy

## Abstract

We aimed to determine the diagnostic yield and outcome of patients receiving antithrombotic drug therapy subjected to small bowel capsule endoscopy (SBCE) for the investigation of small bowel bleeding (SBB). A multicenter retrospective analysis of collected data from all patients undergoing SBCE for the investigation of SBB from March 2003 to June 2023 was performed. The diagnostic yield of SBCE was defined as the detection of positive findings that could explain the cause of the patient’s bleeding. Rebleeding was defined as evidence of bleeding within 1 year after the index episode. During the study period, 8401 patients underwent SBCE for SBB investigation. Bleeding lesions were detected in 1103/2535 (43.5%) antithrombotic users, compared to 1113/5866 (18.9%) in nonusers (*p* < 0.00001). Following capsule endoscopy, a therapeutic intervention was possible in 390/2216 (17.5%) patients with a bleeding lesion. Rebleeding occurred in 927 (36.5%) of antithrombotic users (36.5%), compared to 795 (13.5%) of nonusers (13.5%, *p* < 0.00001). Both the diagnostic yield of SBCE and the rebleeding rates were higher in patients with SBB receiving antithrombotics. Therapeutic intervention was possible in a real-world setting only for a minority of patients with positive findings.

## 1. Introduction

Small bowel bleeding (SBB) is defined as bleeding in the gastrointestinal (GI) tract between the ampulla of Vater and the ileocecal valve. It is suspected when a patient presents with GI bleeding but has negative upper and lower endoscopy findings and it can be presented as overt or occult bleeding. The term “small bowel bleeding” has replaced the previously used term “obscure gastrointestinal bleeding”, now reserved for patients not found to have a source of bleeding even after the performance of small bowel evaluation [1]. The European Society of Gastrointestinal Endoscopy (ESGE), in its recently updated published guidelines recommends small bowel capsule endoscopy (SBCE) as the first-line examination, before consideration of other endoscopic and radiological diagnostic tests, for suspected SBB, given its excellent safety profile, patient tolerability, and the potential to visualize the entire small bowel mucosa (strong recommendation, moderate quality evidence) [1].

Capsule endoscopy, also known as wireless capsule endoscopy or video capsule endoscopy, is a gastrointestinal study that uses a pill camera to take images of the intestinal lumen. The first capsule endoscopy was performed in 1999, and the US Food and Drug Administration approved its use in the United States in 2001. The M2A capsule (mouth to anus) was the first available pill camera and was eventually re-named as PillCam SB (small bowel). The capsule is ingested and transmits images at 2 to 6 frames per second over the course of 8 to 12 h, until the battery expires. It generates 512 by 512-pixel, high-resolution images that allow detailed inspection of the gastrointestinal mucosa [2].

The diagnostic yield of SBCE in patients investigated for SBB ranges from 55% to 70% [3,4,5,6], mainly depending on the time interval between the bleeding episode and the performance of the test [7]. The use of antithrombotics, including antiplatelet agents and anticoagulants, is associated with upper and lower GI bleeding; thus, a higher rate of positive findings is reasonable to expect when patients receiving these drugs undergo SBCE for the investigation of SBB [8,9]. However, the results of published studies concerning the impact of antithrombotics on the SBCE diagnostic yield remain conflicting [10,11,12,13,14]. Furthermore, the clinical outcome of patients under antithrombotics who undergo SBCE for the investigation of SBB has not been well established.

Given the above uncertainties, we aimed to investigate the diagnostic yield of SBCE in SBB patients receiving antithrombotics and to describe the clinical outcome, including the rate of therapeutic intervention and the rebleeding rate, in this specific population.

## 2. Materials and Methods

### 2.1. Study Design

This was a multicenter retrospective study in which we analyzed collected data from consecutive patients subjected to SBCE for the investigation of SBB. Five gastroenterology departments participated in the study, all considered as referral centers for capsule endoscopy in Greece. Data concerning consecutive patients subjected to SBCE between March 2003 and June 2023 (a 20-year study period) were retrieved and analyzed accordingly. Patients receiving antithrombotic treatment, namely antiplatelets or anticoagulants during the bleeding episode were identified. The clinical parameters of the patients including age, gender, and the presence of comorbidities were recorded. Comorbidities, such as a history of hypertension, diabetes mellitus, chronic kidney disease, liver cirrhosis, ischemic heart disease, and hematologic disease were noted. Furthermore, apart from the use of antithrombotics, the use of nonsteroidal anti-inflammatory drugs (NSAIDs), and proton pump inhibitors (PPIs) were also recorded. Capsules in patients with known Crohn’s disease were excluded from the analysis. The study protocol conformed to the principles of the Declaration of Helsinki and was approved by the Institutional Review Boards of the participating hospitals [Gastroenterology Department, Evangelismos General Hospital: 462/25.10.2023; Gastroenterology Department, Ioannina: 121/08.09.2023; Gastroenterology Department, NIMTS: 43/10.07.2023; Gastroenterology Department, 401 Military Hospital: 99/02.10.2023; Gastroenterology Department, Red Cross Hospital: 104/01.11.2023]. Informed consent was waived because of the retrospective nature of the study and the analysis used anonymous clinical data.

### 2.2. Definitions

SBB was defined as bleeding that persists or recurs despite a negative esophago–gastro–duodenoscopy and colonoscopy. SBB was further subclassified into two types: (i) overt bleeding characterized by the passage of visible blood (i.e., melena or hematochezia) and (ii) occult bleeding characterized by recurrent iron deficiency anemia or positive fecal occult blood testing.

Rebleeding was defined as evidence of bleeding: (i) The evidence of melena or hematochezia; (ii) A documented fall in hemoglobin greater than 2 g/dL, after exclusion of other causes of anemia, within 1 year after the index episode in cases of overt SBB or within 1 year after the SBCE testing in patients with occult SBB.

The diagnostic yield of SBCE was defined as detection of positive findings that could explain the cause of the patient’s bleeding. For this reason, the findings were analyzed according to the classification proposed by Saurin et al. and only P2 lesions, i.e., highly relevant findings, were considered positive [15]. Therefore, positive findings included mucosal breaks (mucosal ulcers or multiple erosions), angiodysplasias/angioectasias, and small bowel polyps or tumors with ulcer or bleeding. Less relevant (P1) lesions with uncertain bleeding potential, such as isolated erosions or red spots, venous ectasias and small intact polyps or submucosal (nonerosive) tumors, as well as P0 lesions (i.e., no lesions or findings without any bleeding potential), were considered as negative findings. The location of SB lesions was estimated by transit-time indices, as per standard practice.

### 2.3. Capsule Procedure

SBCE was performed using a PillCam SB1 or SB2; the former was substituted by the latter following its market release (Given Imaging, Ltd., Yoqneam, Israel). Patients fasted for 12 h and received 2 L of polyethylene glycol the evening before swallowing the capsule [16]. After the capsule ingestion, fluids were allowed 2 h later and a light meal 4 h later. After 8–10 h the sensor array and recording device were removed and the digital image stream was reviewed and interpreted the following day. Images were analyzed on a Rapid 6.5 workstation using the Rapid Reader 6 software program (software and workstation from Given Imaging Ltd.).

### 2.4. Endpoints

The study endpoints are described below:

1. To assess the diagnostic yield of SBCE among patients presenting with SBB.
2. To assess the therapeutic intervention rate in patients with positive findings at SBCE.
3. To assess the rebleeding rate in patients with SBB undergoing SBCE.

For each of the study endpoints, comparisons were performed between users and nonusers of antithrombotics.

### 2.5. Statistical Analysis

Age was normally distributed using the Kolmogorov–Smirnov test; expressed as mean ± standard deviation (SD) and compared using the Student’s *t*-test. The categorical data were expressed as rates with percentages and compared using the Chi-squared test or Fisher’s exact test (when the expected cell frequency was <5). All tests were two-tailed and *p*-values < 0.05 were considered to indicate statistical significance. All the statistical analyses were performed using the JMP Pro for Windows software program (version 17).

## 3. Results

During the study period, a total of 8401 patients were subjected to SBCE for the investigation of SBB in the participating hospitals (male/female: 4360/4041, mean age ± SD: 52.6 ± 27.3 years). The capsule reached the caecum, and the entire small bowel was examined in 7589 patients (90.3%). The clinical characteristics of the study population are presented in Table 1. Hypertension was the most common comorbidity, followed by ischemic heart disease and diabetes mellitus.

**Figure 1:**
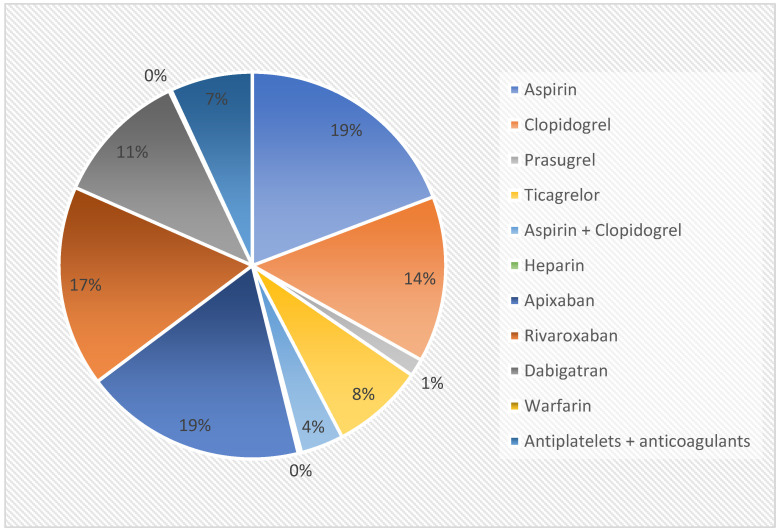
Antithrombotic drugs used by our study population.

Among the (8401) patients, there were 2535 (30.2%) receiving antithrombotic treatment, including antiplatelets, anticoagulants or both. More precisely, among the 2535 patients receiving antithrombotics, there were 1042 (41.1%) receiving antiplatelets (aspirin = 531, clopidogrel = 348, prasugrel = 41, ticagrelor = 212, and aspirin + clopidogrel = 101), 1302 (51.4%) receiving anticoagulants (heparin = 7, apixaban = 512, rivaroxaban = 465, dabigatran = 312, warfarin = 6), and 191 (7.5%) patients that were receiving both. The antithrombotic drugs used by the study population are presented in Figure 1. Among the 8401 patients there were also 4937 receiving PPIs (58.7%).

The clinical presentation of SBB can be seen in Table 2. Most patients were investigated for occult bleeding (n = 6012, 71.5%), namely iron deficiency anemia (n = 4396, 52.3%).

Findings seen on SBCE are presented in Table 3. P0 lesions were detected in 3976 (47.3%) patients, whereas P1 lesions were noted in 2209 (26.3%). Finally, significant (P2) lesions were detected in 2216 (26.4%) patients. Among them, the most common findings were angiodysplasias found in 1459 (65.8%) patients, followed by mucosal breaks (n = 582, 26.3%) and small bowel tumors (n = 175, 7.9%). The P2 lesions were detected in the proximal part of the jejunum in 676 (30.5%) patients, in the distal part of the jejunum in 334 (15.1%) patients, in the proximal part of the ileum in 531 (23.9%) patients, and in the distal part of the ileum in 675 (30.5%) patients.

Positive findings, i.e., P2 lesions seen via SBCE concerning patients either receiving antithrombotics or not, are depicted in Table 3. From the 2535 patients on antithrombotics, P2 lesions were seen in 1103 patients (43.5%), whereas from the 5866 patients not on antithrombotics, P2 lesions were seen in 1113 patients (18.9%). The difference is statistically significant (*p* < 0.00001). The detection rate of significant (P2) lesions was 469/1107 (42.4%) in users of antiplatelets, 444/1008 (44.5%) in users of anticoagulants, and 190/420 (45.7%) in those using both antiplatelets and anticoagulants, yielding no statistically significant difference (*p* = 0.54).

Among the 1103 patients under antithrombotic treatment in whom P2 lesions were detected, a therapeutic intervention was possible in 207 (18.7%). Concerning the 1113 patients not receiving antithrombotics and in whom P2 lesions were found, a therapeutic intervention was possible in 183 (16.4%) (*p* = 0.15). Overall, a therapeutic intervention was possible in 390 patients, corresponding to 17.5% (390/2216) of those found to have a P2 lesion and 4.6% (390/8401) of the whole study population. The therapeutic intervention consisted of endoscopic intervention, such as the application of argon plasma coagulation (APC), a metal clip or epinephrine injection, or a combination of the aforementioned methods, performed in 307 patients (78.7%), whereas a surgical intervention was undertaken in the remaining 83 patients (21.3%). Endoscopic intervention was possible using either a push or double-balloon enteroscope, whereas, in some cases, a conventional colonoscope was used in order to approach lesions in the third or fourth part of the duodenum or in the terminal ileum.

Overall, rebleeding was noted in a total of 1722 (20.5%) patients. The rebleeding rate was 927/2535 (36.5%) in users of antithrombotics and 795/5866 (13.5%) in patients not receiving these drugs (*p* < 0.00001). There was no statistically significant difference concerning the rebleeding rate among patients receiving antiplatelets (383/1107; 34.6%), anticoagulants (380/1008; 37.7%), or both (164/420; 39.1%) (*p* = 0.17).

## 4. Discussion

The present study demonstrates that, in cases of SBB, significant lesions explaining bleeding episodes can be detected in 26.4% of patients undergoing SBCE. The most common findings were angiodysplasias, noted in 65.8% of patients with positive SBCE. The identification of positive findings and therefore the diagnostic yield of SBCE were significantly higher in patients receiving antithrombotics (43.5% vs. 18.9%, *p* < 0.00001). Therapeutic intervention was possible in a minority of patients with positive SBCE (18.7%), whereas rebleeding was more frequent in patients receiving antithrombotics (36.5%) compared to those who did not (13.5%, *p* < 0.00001).

Occult bleeding, presenting as iron deficiency anemia, was the most common indication for SBCE in both patients receiving antithrombotics (44.5%) and those who were not (55.7%). However, the proportion of patients undergoing SBCE due to clinically overt bleeding was significantly higher among users of these drugs (38% vs. 24.2%, *p* < 0.0001).

With the aging population and the more widespread use of antithrombotics, an increasing number of patients receiving anticoagulants or antiplatelet agents will be subjected to SBCE for the investigation of SBB. It is well known that aspirin can affect the small bowel mucosa and cause mucosal damage, erosions and ulcers, potentially leading to SBB [17,18]. On the other hand, the effect of antiplatelet agents on small bowel mucosa has been debated in the existing literature. A retrospective single-center study published in 2014 and including 274 consecutive SBCE examinations performed over a 7-year period could not detect any correlation between antiplatelet drug use and the identification of P2 lesions in patients with SBB [19]. Similarly, in another study including 181 patients, the rate of SBCE positivity among antithrombotic drug users was significantly higher in those with overt bleeding as compared to those with occult bleeding (45% vs. 16%, *p* = 0.014), whereas there was no significant difference concerning the respective rates among antithrombotic nonusers (27% vs. 26%, *p* = 1.0) [20]. On the contrary, in a multicenter, hospital-based, case-control study including 400 patients, thienopyridine (AOR, 3.2; *p* = 0.015) and anticoagulant use (AOR, 4.3; *p* = 0.028) were independently associated with SBB [21]. Furthermore, in a retrospective review of chronic users of antithrombotics who underwent SBCE for suspected SBB, the combined administration of aspirin and thienopyridine significantly exacerbated small bowel damage resulting in more erosions and ulcers [22]. Finally, a systematic review and meta-analysis evaluated the effect of antithrombotic drugs on SBCE findings, showing that antithrombotic treatment was associated with an increased prevalence of positive findings compared to antithrombotic nonusers [OR 1.98 (95% CI 1.34–2.93); *p* = 0.0006] [23]. This effect did not differ between antiplatelet and anticoagulant treatments [OR 2.22 (95% CI 1.28–3.84); *p* = 0.005 and 2.53 (95% CI 1.66–3.87); *p* < 0.0001, respectively]. Antithrombotic use over no use was neither associated with overt [OR 1.17 (95% CI 0.51–2.66); *p* = 0.71] nor with occult [OR 0.86 (95% CI 0.38–1.95); *p* = 0.71] bleeding pattern [23]. Our results are in accordance with the above data, since SBCE revealed significantly more bleeding lesions in patients receiving antithrombotics as compared to those who did not (43.5% vs. 18.9%, *p* < 0.00001).

There are several contraindications to capsule endoscopy. Since the procedure requires patient participation, individuals with dementia are usually poor candidates. Swallowing disorders may cause difficulty in ingesting the capsule. There is a relative contraindication in patients with cardiac pacemakers, defibrillators, or left ventricular assist devices due to a concern for possible interference between the capsule and the cardiac devices. However, there has never been a reported case of cardiac device malfunction due to capsule endoscopy. Furthermore, studies have demonstrated that there is no interference with these cardiac devices. Pregnant women should not have capsule endoscopy since there are no studies on the safety of capsule endoscopy in this patient population. The risk of capsule retention is greatest in patients with known or suspected strictures, fistulas, and obstructions. Capsule endoscopy is contraindicated in these patients due to the risk of worsening obstructions or causing obstructions. If capsule endoscopy is necessary for a patient at risk for retention, an agile patency capsule can be ingested. An agile patency capsule is a radiopaque capsule without video capabilities. Thirty hours after ingestion, a plain abdominal film or hand-held scanner can be used to determine the passage of the capsule. This determines if it is safe to proceed with video capsule endoscopy. The video capsule can also be retained due to achalasia, esophageal diverticula, esophageal strictures, or pyloric stenosis. Gastroparesis may slow the mobilization of the capsule [24].

Capsule retention can occur in 1.3% to 1.4% of patients undergoing capsule endoscopy and is the most common complication. Capsule retention is usually asymptomatic and diagnosed 2 weeks after capsule ingestion via abdominal plain film x-ray [25].

In the future, the implementation of artificial intelligence (AI) might solve the triage of normal images or even the characterization of abnormalities. Deep learning models have been suggested for blood identification, demonstrating a significantly elevated sensitivity, specificity, and precision, exceeding 98%. The application of AI using a convolutional neural network (CNN) exhibited automatic detection of angioectasias with remarkable sensitivity (98.8%) and specificity (98.4%). Nonetheless, further prospective clinical studies are imperative to validate these promising outcomes and despite the advancements and accomplishments to date, numerous gaps and uncertainties persist regarding the performance of AI. Until AI is thoroughly validated and deemed entirely trustworthy, the responsibility for interpreting images will continue to rest with humans, specifically a physician possessing training and expertise in capsule endoscopy reading [26].

One of the disadvantages of SBCE is its inability for therapeutic intervention. Although capsule endoscopy has revolutionized the examination of the small bowel mucosa, therapeutic intervention requires a push or a single- or double-balloon enteroscopy whenever a lesion is identified in SBCE [24]. The double-balloon enteroscopy (DBE) system, including an enteroscope, an overtube, and two inflatable balloons—one at the distal end of the endoscope and the other attached to the overtube—works using the “pull-and-push” technique; the distal balloon helps anchor the endoscope in place, preventing it from slipping out, while the proximal balloon helps advance the endoscope deeper into the small bowel. Two methods of insertion are possible, either ante-grade—via the mouth, passing the esophagus and the stomach first—or retrograde, via the anus and colon [27]. The single-balloon enteroscopy (SBE) technique was designed to simplify the push-and-pull method and relies on the use of a single balloon attached to the tip of the overtube, resulting in a reduced setup time and more user-friendly balloon control panel [27]. Enteroscopy plays a pivotal role in the therapeutic approach of patients with SBB, having a high clinical impact according to published data [25,26]. Thus, SBCE could be used as a primary diagnostic modality aiming to identify the bleeding lesion, followed by treatment of the lesion with enteroscopy [27]; however, there are at least three major drawbacks to this dual-modality approach: (i) despite advances in SBCE technology, accurate localization of detected lesions within the featureless structure of the small bowel remains challenging; (ii) double-balloon enteroscopy, which is the only examination that has the potential to visualize the entire small bowel mucosa, remains a demanding technique operated by experts and it is not widely available at every institution (indeed, double-balloon enteroscopy was only available in one out of the five participating centers in our study); and (iii) many lesions, such as angiodysplasias, noted in SBCE are not subsequently detected during enteroscopy, due to their intermittent bleeding potential. Congruently, this study determined that only a minority (17.5%) of patients with positive SBCE underwent a therapeutic intervention, further decreased to 4.6% if one takes into account the whole study population of patients with SBB undergoing investigation with SBCE.

Rebleeding is often observed in patients undergoing SBCE for SBB [28,29]. According to a previous study, the overall rebleeding rate appeared to be 19.0% during a mean (±SD) follow-up of 38.7 ± 26.4 months [30]. However, concurrent antithrombotic treatment was associated with higher odds for rebleeding compared to no treatment [OR 2.53 (95% CI 1.46–4.37); *p* = 0.0009] [22]. Similarly, antithrombotic users with overt SBB and positive findings at SBCE had a greater risk of rebleeding in comparison to those with negative SBCE (50% vs. 5.9%, *p* = 0.011) [20]. According to our results, there was a statistically significant difference concerning the rebleeding rate between users and nonusers of antithrombotic drugs (36.5% vs. 13.5%, respectively). This is, at least partly, explained by the fact that antithrombotics have the potential to exacerbate bleeding from small bowel vascular lesions, such as angiodysplasias, representing the lesions most commonly described in patients using these drugs [31,32].

Angiodysplasias were by far the most common finding in our study population. Gastrointestinal angiodysplasias, also called angioectasias, are vascular malformations composed of dilated and tortuous arterial or venous capillaries, which are usually smaller than 5 mm in diameter and located in the mucosal and submucosal layers of the gastrointestinal tract. The prevalence of agiodysplasias is difficult to determine because most of them are asymptomatic. They are incidentally found in 1% to 5% of patients who underwent endoscopic studies and remain asymptomatic in a period of 3 years of follow-up. These vascular lesions are mostly detected in patients older than 60 years, so it is thought that they have a degenerative component [33]. Angiodysplasias can be found in any segment of the digestive tract; however, they are more frequent in the small bowel (57–80%), particularly in the proximal segment, followed by the colon (44%) and the stomach (32%). It is worth noting that angiodysplasias are located in more than one segment of the digestive tract in 60% of cases [34]. Known risk factors for gastrointestinal angiodysplasias are age > 60 years, chronic obstructive pulmonary disease, aortic stenosis, ischemic heart disease, liver cirrhosis, venous thromboembolism and the use of antithrombotic drugs. The pathophysiology of angiodysplasias is not well known. The finding of non-bleeding angiodysplasias by endoscopy in patients with bleeding confronts the clinician with the dilemma of the bleeding responsibility of such lesions. Most authors suggest that in the absence of other evident actively bleeding sources, the responsibility of angiodysplasias should be accepted [35]. Meanwhile anticoagulation is indicated for a variety of highly prevalent cardiovascular conditions, including venous thromboembolism, atrial fibrillation, and mechanical heart valve prostheses. While anticoagulants substantially decrease the risk of thromboembolism, major bleeding remains their most important complication. Nearly half of oral anticoagulant associated major bleeding arises from the gastrointestinal tract, and angiodysplasias are to be blamed when the small bowel is the site of the bleeding episode [36].

A major strength of this study is the large number of included patients over a long 20-year study period. Due to the multicenter design, we were able to analyze data from 8401 patients undergoing SBCE for the investigation of SB. To the best of our knowledge, this represents the largest database reported so far. Obviously, our study has several limitations, including its retrospective nature and the fact that not all centers had the potential for therapeutic intervention in patients with positive findings at SBCE. Furthermore, we do not have specific information regarding the detailed description of antithrombotic drugs or information as to how many patients were still taking their antithrombotic agents during the capsule endoscopy. Therefore, the relevant information reported in our study regards the use of antithrombotics only during the bleeding episode. In addition, specific information as to how many patients had aortic stenosis or other conditions specifically associated with angioectasias was also not recorded. Lastly, there is a lack of clinical information regarding the final diagnosis, and there are no data regarding the laboratory parameters, in particular the coagulation panel and/or platelet counts of our study population, which may have affected the GI bleeding since most of the patients included in the analysis were referred to us from other centers and such information was not available.

Nevertheless, we believe our data provide relevant insights for practicing clinicians, pointing out that patients on antithrombotics warrant immediate and meticulous small bowel examination, since both the possibility of significant findings and the possibility of rebleeding if these lesions remain untreated are high. However, it has to be noted that the therapeutic intervention rate appears to be low, given the fact that capsule endoscopy cannot provide accurate localization of the bleeding lesions and double-balloon enteroscopy is not available in most centers.

In summary, most patients subjected to SBCE for the investigation of SBB present with occult bleeding and, more specifically, iron deficiency anemia. Significant lesions that can explain the bleeding episode can be found in approximately ¼ of those subjected to the test, and angiodysplasias are by far the most common finding. Bleeding lesions are mostly found in the proximal part of the jejunum and the distal part of the ileum. Patients receiving antithrombotics not only present with bleeding lesions more commonly than those not receiving these drugs but also rebleed more often. Therapeutic intervention is possible in less than 20% of those patients found to have significant bleeding lesions. Future advancements in SBCE logistics could possibly help us better localize the bleeding lesions, and more widespread use of interventional enteroscopy could enhance our therapeutic potential in all patients, but especially in those receiving antithrombotics.

In conclusion, the diagnostic yield for detecting lesions with significant bleeding potential is higher in patients taking antithrombotics, highlighting the importance of performing SBCE as part of the routine evaluation for SBB in this specific subset of patients. Interestingly, both anticoagulants and antiplatelets contributed to the examination’s higher positivity rate. Furthermore, rebleeding occurred more frequently among users of these drugs, emphasizing that users of antithrombotics represent a high-risk population for recurrent SBB, likely to benefit from individualized surveillance strategies. Our study, based on the largest dataset analyzed so far, provides strong evidence on the impact of antithrombotic treatment on SBB, potentially allowing for more accurate patient selection for investigation and therapeutic intervention, with final improvement of clinical outcomes.

## Figures and Tables

**Table 1:** Clinical characteristics of the study population.

	Whole Population	Patients Receiving Antithrombotics	Patients Not Receiving Antithrombotics	*p* Value
Age, mean (SD), years	52.6 ± 27.3	56.1 ± 29.1	49.9 ± 25.4	<0.0001
Sex, males/females, number of patients	4360/4041	1302/1233	3058/2828	0.61
Comorbidities, n (%)				
Hypertension	2854 (33.9%)	804	2050	0.006
Diabetes mellitus	1982 (23.5%)	(31.7%)	(34.9%)	0.05
Chronic kidney disease	1077 (12.8%)	631	1351	0.0009
Liver cirrhosis	233 (2.8%)	(24.9%)	(23.0%)	<0.0001
Ischemic heart disease	2106 (25.1%)	278	801	<0.0001
Hematologic disease	349 (4.2%)	(10.9%)	(13.6%)	<0.0001

**Table 2:** Clinical presentation of small bowel bleeding.

	Whole Population (n = 8401)	Patients on Antithrombotics (n = 2535)	Patients without Antithrombotics (n = 5866)	*p* Value
Overt Bleeding, n (%)	2389 (28.4)	963 (38)	1426 (24.2)	<0.0001
Melena, n (%)	1733 (20.6)	647 (25.5)	1086 (18.5)	<0.0001
Hematochezia, n (%)	656 (7.8)	316 (12.5)	340 (5.8)	<0.0001
Occult bleeding, n (%)	6012 (71.5)	1572 (62)	4440 (75.7)	<0.0001
Iron deficiency anemia, n (%)	4396 (52.3)	1127 (44.5)	3269 (55.7)	<0.0001
Positive fecal occult blood testing, n (%)	1616 (19.2)	445 (17.6)	1171 (20)	0.01

**Table 3:** Findings of small bowel capsule endoscopy.

	Whole Population (n = 8401)	Patients on Antithrombotics (n = 2535)	Patients without Antithrombotics (n = 5866)	*p* Value
P0 lesions, n (%)	3976 (47.3%)	723 (28.5)	3253 (55.4)	<0.0001
P1 lesions, n (%)	2209 (26.3%)	709 (27.9)	1500 (25.6)	0.02
Isolated erosions or red spots	1208 (14.3%)	356 (14.1)	852 (14.5)	0.60
Venous ectasias	864 (10.3)	302 (11.9)	562 (9.5)	0.001
Small polyps or submucosal tumors	137 (1.6%)	51 (2.0)	86 (1.4)	0.07
P2 lesions, n (%)	2216 (26.3%)	1103 (43.5)	1113 (18.9)	<0.0001
Mucosal breaks	582 (6.9%)	241 (9.5)	341 (5.8)	<0.0001
Angiodysplasias	1459 (17.4%)	781 (30.8)	678 (11.5)	<0.0001
Small bowel tumors	175 (2.1%)	81 (3.2)	94 (1.6)	<0.0001

## Data Availability

Material available upon request to interested researchers.
